# Long-Term Performance and Stability of Interlayer-Free Mesoporous Silica Membranes for Wetland Saline Water Pervaporation

**DOI:** 10.3390/polym14050895

**Published:** 2022-02-24

**Authors:** Muthia Elma, Muhammad Roil Bilad, Amalia Enggar Pratiwi, Aulia Rahma, Zaini Lambri Asyyaifi, Hairullah Hairullah, Isna Syauqiah, Yulian Firmana Arifin, Riani Ayu Lestari

**Affiliations:** 1 Chemical Engineering Department, Engineering Faculty, Lambung Mangkurat University, Banjarbaru 70714, Indonesia; isna_tk@ulm.ac.id (I.S.); ra.lestari@ulm.ac.id (R.A.L.); 2 Materials and Membranes Research Group (M2ReG), Lambung Mangkurat University, Banjarbaru 70714, Indonesia; aepratiwi@mhs.ulm.ac.id (A.E.P.); arahma@mhs.ulm.ac.id (A.R.); zaini.labri.assyaifi@gmail.com (Z.L.A.); 1920834310001@mhs.ulm.ac.id (H.H.); 3 Faculty of Integrated Technologies, Universiti Brunei Darussalam, Gadong BE1410, Brunei; 4 Environmental Science Doctoral and Postgraduate Program, Lambung Mangkurat University, Banjarmasin 70123, Indonesia; 5 Professional Engineer Education Study Program, Lambung Mangkurat University, Banjarbaru 70714, Indonesia; y.arifin@ulm.ac.id; 6 Civil Engineering Study Program, Lambung Mangkurat University, Banjarbaru 70714, Indonesia

**Keywords:** desalination via pervaporation, interlayer-free silica membrane, wetland saline water desalination

## Abstract

Wetland water is an alternative water resource around wetland areas. However, it is typically saline due to seawater intrusion and contains high natural organic matter (NOM) that is challenging to treat. This study evaluated the stability of interlayer-free mesoporous silica matrix membranes employing a dual acid–base catalyzed sol–gel process for treatment of saline wetland water. The silica sols were prepared under a low silanol concentration, dip-coated in 4 layers, and calcined using the rapid thermal processing method. The membrane performance was initially evaluated through pervaporation under various temperatures (25–60 °C) using various feeds. Next, the long-term stability (up to 400 h) of wetland saline water desalination was evaluated. Results show that the water flux increased at higher temperatures up to 6.9 and 6.5 kg·m^−2^·h^−1^ at the highest temperature of 60 °C for the seawater and the wetland saline water feeds, respectively. The long-term stability demonstrated a stable performance without flux and rejection decline up to 170 h operation, beyond which slow declines in water flux and rejection were observed due to fouling by NOM and membrane wetting. The overall findings suggest that an interlayer-free mesoporous silica membrane offers excellent performance and high salt rejection (80–99%) for wetland saline water treatments.

## 1. Introduction

Generally, Indonesia has considerable water resource potential from river, basin, lake, seawater, and wetland water. However, high-quality water scarcity is still common in remote areas, like in South Kalimantan, despite having large water wetland water resources [1]. Wetland water is still used for household needs by rural communities [2]. It has unique characters, such as brown color, low pH, and high organic matter, especially natural organic matter (NOM). During the hot season, the salt concentration of wetland water is high [3], caused by seawater intrusion into rivers during the rainy season that evaporates during the hot season. Therefore, desalination of saline peatland is necessary before being used.

Membrane technology has long been used for water and wastewater treatment. Most of the membrane materials for reverse osmosis desalination are polymeric. Inorganic membranes have also been widely reported for water separation [4]. The amorphous silica membranes have demonstrated a good performance [5] and have a great molecular sieving property. Silica-based membranes have excellent molecular sieving properties and a simpler fabrication process. Their structures have pore sizes in the range of 3–5 Å on the order of the kinetic diameter of the water molecule (dk = 2.6 Å), thus ideal to hinder the passage of hydrated salt ions (e.g., Na^+^: dk = 7.2 Å and Cl^−^: dk = 6.6 Å). On the other hand, complex chemistry and difficulty in controlling the structure and pore size distribution is the main challenge of developing polymeric membranes for pervaporation-based desalination [6]. High-performance inorganic membranes can be produced through a simple sol–gel process. However, the main issue of a silica membrane is weak hydro-stability [7] when in contact with water molecules. Hydro-stability is influenced by silanol groups (Si-OH) that typically form pores with a size of <1 nm [8]. The problem occurs if the hydrophilic silanol reacts with the feed water that enlarges the pore size [9]. In addition, the silica structures also collapse when reacting with water molecules [10].

Maintaining the pore size of silica-based inorganic membrane is the key to raising great performance and stability for water desalination. The sol–gel method has proven reliable for fabricating the typical interlayer-free mesoporous silica membrane [11]. The use of catalysts is also important during the sol–gel process. De Vos, et al. [12] fabricated silica membranes by the acid-catalyzed sol–gel method and produced molecular sieving (micropores) but showed a low water flux. Base catalyst, in the contrary, forms bigger pores. Therefore, the application of dual catalyst has been employed to optimize the size of silica pores. The previous study employed a dual acid–base catalyst to reduce amount of silanol groups. The first step involved acid catalysis and second step included ammonia base catalysis resulting in a membrane with optimum mesoporous silica matrices. The application of the rapid thermal processing (RTP) for calcination method is also important to lower the fabrication time and costs [13,14,15].

A few studies reported the stability of a silica-based membrane for saline water desalination by pervaporation. Lin, et al. [16] demonstrated cobalt oxide silica membranes (CoOxSi) used for treating feeds containing multiple salt solutions i.e., 1–15 wt% NaCl. The pervaporation performance was evaluated for 570 h. In other works [9], CTMSS (C6) membranes displayed stable performance over 5–12 h, due to the benefit of the carbonized templating method to improve the hydro-stability of the silica membranes [17]. Previous studies have also demonstrated the long-term stability of silica membrane prepared by the RTP method that is stable for about 100 h of operation for treating wetland saline water [18]. Afterward the water flux decayed gradually by 25% until 250 h. The research and long-term stability evaluation of silica-based membranes for PV is still limited, especially applied to the desalination of wetland saline water.

This study developed and characterized a silica xerogel membrane and evaluated its long-term stability for wetland water desalination via pervaporation. After fabrication, the membrane material was characterized to investigate the structure of silica network bonding through scanning electron microscopy (SEM), Fourier transform infrared (FTIR) spectroscopy, Brunauer, Emmett and Teller (BET) analysis, and the N_2_ sorption test. The desalination performance was later evaluated by pervaporation of different feeds under different feed temperatures. Finally, long-term stability tests were conducted to treat actual wetland saline water.

## 2. Materials and Methods

### 2.1. Materials and Thin Film Fabrication

Silica sol was synthesized by the dual acid–base sol–gel method. Tetraethyl orthosilicate (TEOS, 99.0%, Sigma-Aldrich, St. Louis, MO, USA) as a precursor of silica was added dropwise into ethanol (EtOH, 99%) solution and stirred for 5 min in a cold bath at 0 °C to avoid a hydrolysis reaction. Next, 0.00078 N HNO_3_ (Merck) was added dropwise refluxed for 1 h at 50 °C. It was then diluted by dropwise addition of 0.0003 N NH_3_ (Merck) into the silica sols and stirred for 2 h. Finally, the silica sol was obtained with the final molar ratios of TEOS:EtOH:H_2_O:NH_3_:HNO_3_ being 1:38:5:0.0753:x, where x was varied according to the HNO_3_ concentrations from 0.195 to 2 for preparation of sol at pH 1.45 to 9 ± 0.1. The sol was dried in an oven for 24 h and then ground as xerogels for characterization.

Silica thin films were coated four times directly onto macroporous alumina substrates (α-Al_2_O_3_ tubular support, Ceramic Oxide Fabricators, Bendigo, Australia) via the dip-coating process with a dwelling time of 2 min and dipping and withdraw rates of 10 and 5 cm min^−1^, respectively. Subsequently, the membrane layers were dried in an oven then calcined in a furnace for 1 h at 600 °C.

### 2.2. Membrane Characterization

FTIR spectra data were used to capture the functional groups on the silica sol surface. The spectra were collected from FTIR (Bruker Alpha, alpha sample compartment RT-DLaTGS) within the wavelength range of 4000–500 cm^−1^ for a total of 30 scans using an ATR platinum Diamond 1 Relf attachment. The obtained peaks were deconvoluted using Fityk software over the 1300–700 cm^−1^ with approximately 5% error. The Gaussian curve was chosen to fit the peaks. HWHM (the half-width half max) values were consistently fixed for the same deconvolute peaks.

The membrane’s specific surface area and pore size were determined using BET method, and the last point of the isotherm, respectively. The isotherm was analysed with N_2_ sorption at 77 K and 1 bar using the Micromeritic TriStar 3020 instrument. The membrane morphology and thickness were characterized by a SEM (EVO^®^ MA 10).

### 2.3. Desalination Performance and Long-Term Stability

**Figure 1:**
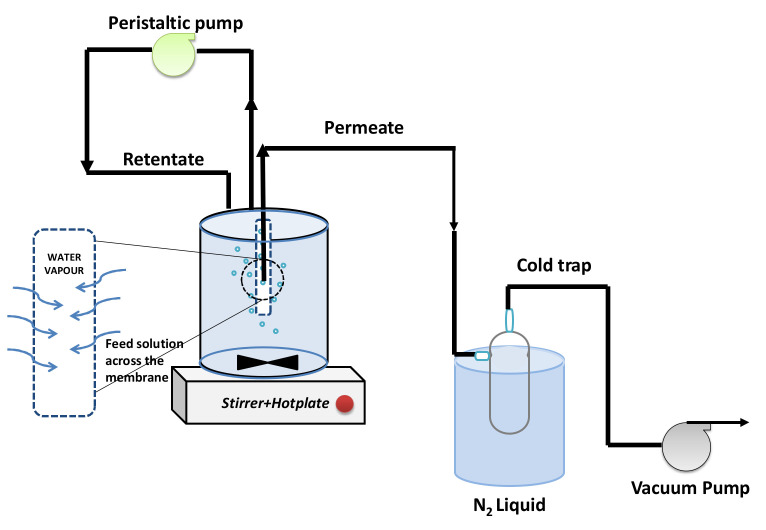
Illustration of the pervaporation experiment set up for desalination.

For desalination tests, the membrane was installed in a pervaporation set-up (Figure 1). One side of the membrane was connected with a vacuum pump to allow a dead-end operation. The other with effective area of 6 cm^2^ was immersed in the feed tank with a volume 0.5 L. The feeds were 3.5 wt%, NaCl (Sigma-Aldrich) or wetland saline water. The tests were done at various temperatures of 25 to 60 ± 2 °C. The feed solution was stirred to minimize concentration polarization. The water flux (*F*, kg m^−2^ h^−1^) and salt rejection (*R*, %) were calculated using Equations (1) and (2).

(1) $$F=m/\left(A\Delta t\right)$$

(2) $$R=\left(C_{f}-C_{p}\right)/C_{f}\times 100\%$$

where *m* is permeate mass (kg) retained in the cold trap, *A* is the surface-active area of the membrane (m^2^), Δ*t* is operation time (h), and *C_f_* and *C_p_* are the feed and permeate concentrations. The salt concentration was estimated from a calibration curve that links it with conductivity data measured by a conductivity meter (OHAUS SF300C-G).

The membrane performances for desalination were evaluated in the short-term using the following feeds: demineralized water (0 wt%), wetland saline water (3.2 wt%), seawater (3.5 wt%), and brine (5% salt) at varied feed temperatures: 25 to 60 ± 2 °C. Finally, long-term stability tests were conducted by treating actual wetland water obtained in both the wet and the dry seasons.

## 3. Results and Discussion

### 3.1. Xerogel Surface Chemistry

**Figure 2:**
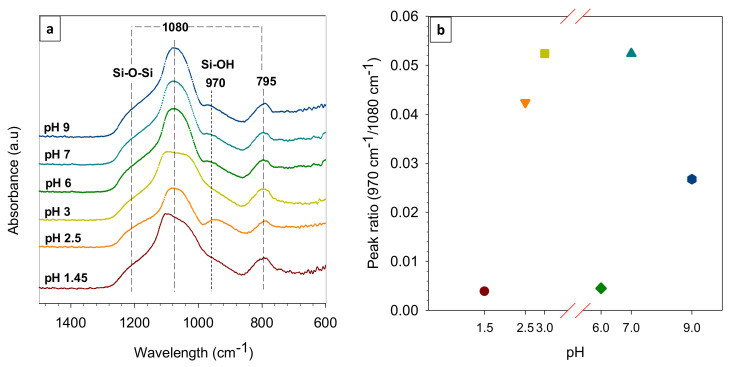
(**a**) FTIR spectra for the xerogels and (**b**) the peak area ratio of the silanol against the siloxane at peaks 970 vs. 1080 cm^−1^.

Figure 2a shows the FTIR spectra of the calcined xerogel samples prepared under all pHs. This characterization helps in identifying available chemical bond near the surface of the sample and provide the indication of the effectiveness of the synthesis process. The spectra profiles suggest that the chemical constituents are similar for all samples. They were vibrational bands in 1400–600 cm^−1^. The intense peaks at 1112, 1080 cm^−1^, and 790 cm^−1^ indicated the presence of siloxane (groups), while the peak at wavelength 970 cm^−1^ was for silanol (Si-OH) bond [19].

The peak area ratio was analyzed to compare the silanol against the siloxane as shown in Figure 2b. It helped to identify the relative abundance of the silanol group that corresponded to a smaller pore size. The ratio increased at a higher pH at a range 1.45 to 3, then decreased at pH 6, and increased again at pH 7 and 9. This behavior can happen due to the pH-dependency of hydrolysis, condensation, and polymerization in the silica sols, as reported earlier [20,21,22]. For pH 6, the finding was quite similar to the previous work [23], in which the ratio decreased to a minimum value for the silica–pectin membrane. If one compared only three variances of pH (6 to 9), the silica sol at pH 6 showed the lowest silanol and the highest siloxane bridge concentration affecting the pore size. The silanol groups likely resulted in smaller pore sizes (micropores) [16], while the mesopores and macropores were formed by the siloxane content [24]. The combination of silanol and siloxane concentration would yield mesoporous or bottleneck pores [25], which was expected to boost the performance in terms of water flux and salt rejection From this finding, further analysis and performance tests were done only for the calcined xerogel prepared at pH 6.

The surface and pore properties of the bulk silica xerogels were analyzed from the N_2_ sorption data shown in Table 1. The silica xerogels prepared at pH 6 showed a tendency as a mesoporous material. There was a space between the adsorption and the desorption lines, indicating the presence of holes. The adsorption saturation was achieved at 0.98–0.4 p/p^o^ as the capillary condensation led to hysteresis. An earlier study [7] also reported the fabrication of silica xerogel that resulted in a mesoporous structure. The RTP method was also found to affect the resulting membrane thickness and the desalination performance. The TRP method promoted condensation leading to siloxane bridges, forming a more robust silica network to resist drying stresses.

The results on the average pore diameter listed in Table 1 confirm the formation of mesoporous membranes with pore sizes between 2 and 50 nm. The thickness of the silica thin films was expected to affect the strength of the silica network. The obtained membrane thickness in this work was thicker than our earlier work [26] due to evaporation of the ethanol solvent that formed a more robust silica bond matrix creating spaces to increase the thickness.

**Table 1:** Surface properties of bulk silica xerogels calcined in air.

Sample Code	BET Surface Area (m^2^·g^−1^)	Pore Volume (10^−6^ m^3^·g^−1^)	Average Pore Diameter (nm)	Thickness (nm)	Ref.
Pure silica (RTP)	272	0.17	2.50	~1000	This work
Pure silica (CTP)	402	0.221	2.70	400	[27]

Table 1 shows that the silica pure xerogels pore volume was slightly higher than the one obtained from the same fabrication method previously [14,27]. The average pore was mesoporous, although the pore of the pure silica membrane was larger than the RTP-based membrane. However, the surface area was significantly different. Membrane prepared from the CTP method resulted in up to 30% higher surface area than the ones produced by the RTP method. However, the finding disagreed with others [14,27] that reported higher surface area for the RTP-based membranes. Unlike this study, they increased the RTP temperature, densifying the silica matrix, depleting the surface area by about 10%. The disagreement could also be explained by the difference in the molar ratio of water used in this work. Previous reports [14,27] compared the water molar ratio, and only used acid catalysts in the sol–gel process. Despite the lower surface area of the developed silica membrane, the acid–base catalysts used in the sol–gel process enhanced the mesoporous structure, beneficial for desalination [28]. Earlier reports [24] showed the formation of a mesoporous membrane structure attributed to the RTP calcination.

### 3.2. Membrane Morphology

**Figure 3:**
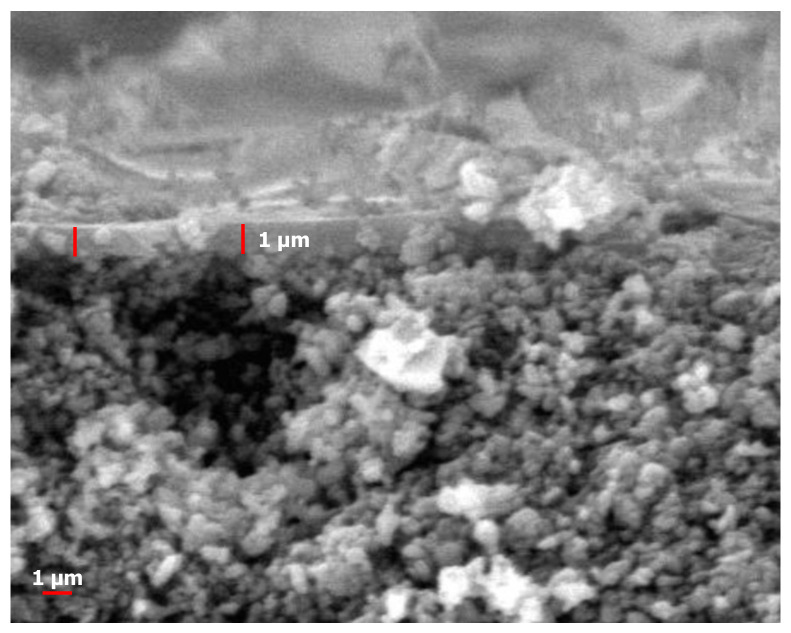
Cross-sectional SEM image of silica membrane in sol pH 6.

Figure 3 shows the SEM images of the prepared pure silica membrane coated directly onto a macroporous alumina substrate. The cross-section image aided in identifying the formation of the coating layer and how it was attached to the alumina support. The cross-section image clearly shows the coating layer atop the support. The surface morphology was rough atop the membrane support due to atmospheric calcination conditions instead of a vacuum, as reported recently [29], to fabricate hollow fiber PVDF-TiO_2_ membranes. The membrane surface morphology affected the water flux. The homogeneous surface without cracks, as shown in Figure 3, contributed to good salt rejection [30]. The presence of cracks should be considered as a membrane defect that allows free flow of feed across the membrane without undergoing any separation.

The thickness of a silica thin film coated on the surface of an alumina support was ~1 µm as shown on Figure 3. It was thinner than the membrane prepared from a TEOS and carbon template-derived banana peel pectin of 2 µm [31]. In addition, this silica top layer was more than 30 times thicker than other silica membranes fabricated earlier [12], since they only employ two coating layers. Generally, silica thin films coated onto γ-Al_2_O_3_ interlayers had pore sizes of much smaller (3–5 nm) than the pores of the α-Al_2_O_3_ support (100–300 nm) [32,33]. Acid-catalyzed silica films were usually coated to avoid cracking on top of silica films [16,34,35]. However, we demonstrated in this work that coating the silica thin film with an acid–base catalyzed directly onto a mesoporous substrate as a support was also possible and—as deduced from long-term stability data—was free from any cracks. The fabrication method could effectively reduce the time and cost of fabricating silica membranes.

### 3.3. Desalination Performance and Long-Term Stability

**Figure 4:**
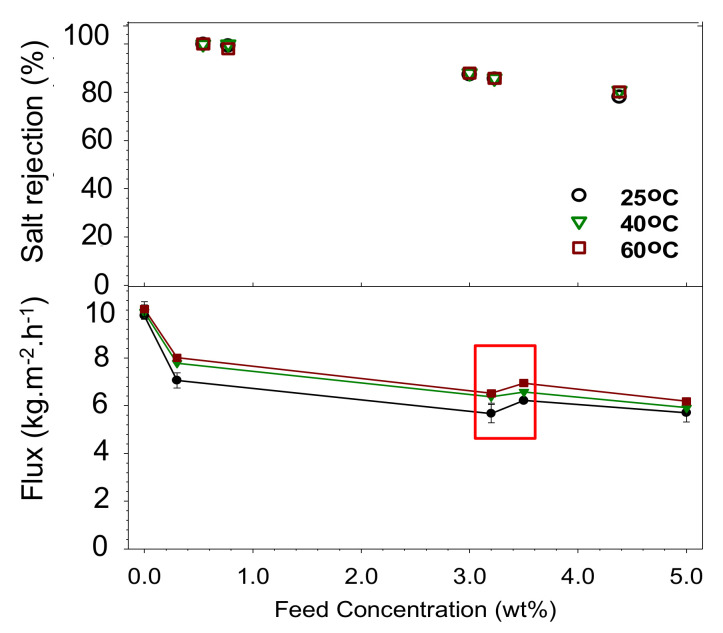
Pervaporation flux and salt rejection of feeds under various salt concentrations and feed temperatures.

The chemical and structural properties of silica-based membranes were tailored to achieve desirable desalination performance represented by high water flux and salt rejection. Figure 4 shows the pervaporation performances of water, wetland saline water (3.2 wt% of salt), and seawater (3.5 wt% of salt) at varied feed temperatures of 25 to 60 ± 2 °C. It shows that the water flux increased at higher feed temperatures, with the opposite trend for the salt rejection. The higher temperature of the feed increased the vapor pressure to drive water vapor permeation [30]. In addition, the pore size distribution was dictated by a combination of micro- and mesopores that affected the water flux and the salt rejection [5,36]. The highest obtained water fluxes were 6.9 kg·m^−2^·h^−1^ and 6.5 kg·m^−2^·h^−1^, and rejections were 85% and 96% obtained at 60 °C of for the seawater and the wetland saline water, respectively. This water flux result was fivefold higher than the membrane pure silica reported earlier [37] for desalination of wetland saline water at feed temperature 60 °C via pervaporation. Figure 4 also shows that the water quality of the permeate for the 5 wt% feed concentration was still lower than the WHO standard of the sodium limits for potable water. The salt rejections of all feeds in multiple feed temperature (25–60 °C) exhibit high values of 80–98%. Both the water flux and salt rejection data suggest that the developed membrane offered good performance in desalination.

The pervaporation performance of the developed membrane was better than in previous reports. A modified silica membrane by metal oxide resulted in 0.3 kg·m^−2^·h^−1^ of water flux for brackish water treatment at room temperature [31]. A hybrid membrane system for seawater desalination reached a water flux of 3.5 kg·m^−2^·h^−1^ and 83% salt rejection at room temperature [38].

The pure silica interlayer-free membrane by CTP showed lower water flux compared to the previous work employing different calcination duration of Elma, Yacou, Costa and Wang [26]. In this work, the RTP took 1 h for calcination of each coating layer, while the referenced work of Elma, Yacou, Costa and Wang [26] took 4 h. The calcination duration affected the robust structure of the resulting silica network.

Another critical phenomenon during the desalination of water is the adsorption of salt ions by the hydrophilic silica surface [27]. Water evaporation left salts to interact with the hydrophilic silica. The silanol groups formed less rigid structures within the silica matrix, which reduced the stability due to the breakdown of the siloxane bridges [39].

Figure 4 (red box) shows the water flux of wetland water was higher than the seawater. Although wetland saline water had a lower salt concentration, the water flux was lower, which can be attributed to a high organic compound known as NOM. The membrane fouling lowered the fluxes of wetland saline water by NOM. NOM is usually represented in UV-254 absorbances, oxidizable organic compound by KMnO_4,_ and dissolved organic carbon (DOC). Haan, et al. [40] reported the concentration of dissolved organic carbon in surface water was estimated commonly by Fluorescence (F) and light absorbance. The high concentration of NOM is the main problem in a wetland water treatment. Some conventional treatments have handled it so far, including coagulation-flocculation, adsorption, and membrane filtration [41,42]. NOM caused membrane fouling that needs to be limited to achieve high system productivity [43,44,45]. Membrane fouling is the major challenge in the membrane filtration system that depleted the flux during operation [46,47].

**Figure 5:**
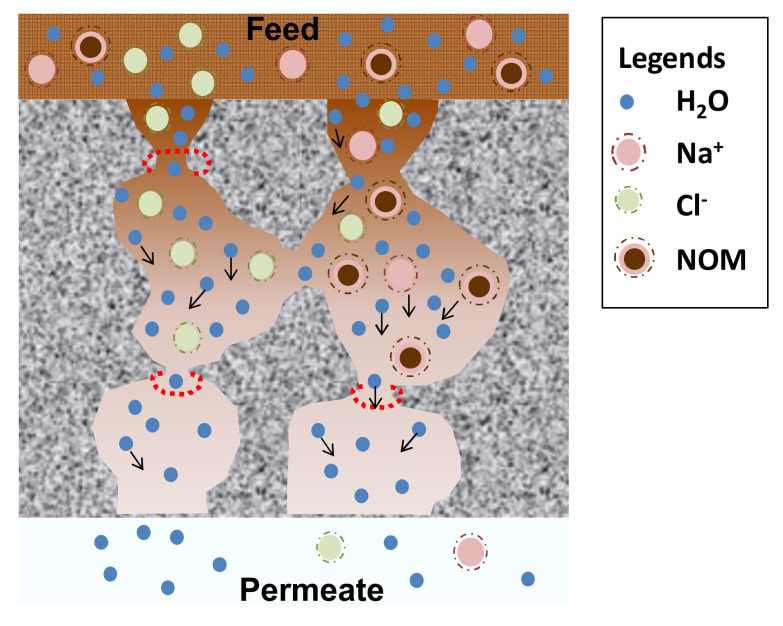
Illustration of membrane fouling phenomenon caused by natural organic matter in wetland water, also showing percolative porous pathway in water desalination.

Figure 5 illustrates the membrane fouling phenomenon by wetland water. It shows how the salts (Na^+^ and Cl^−^ ions), NOM, and water molecules transport across the pathway of bottleneck pores. A high NOM content caused the polarization concentration on the membrane surface. The larger hydrated salt ions blocked or constricted the pore, eventually limiting the pathways for water permeation, reducing the water flux [16,48].

In the context of xerogels, pore-blocking of percolative pathways of silica matrix by the larger hydrated salt ions could also happen. The salt rejection decreased by increasing the salt feed concentration because of salt polarization. For the wetland saline water feed, the NOM compound also increased the salt rejection by forming an additional cake filtration layer.

### 3.4. Long-Term Stability Performance

**Figure 6:**
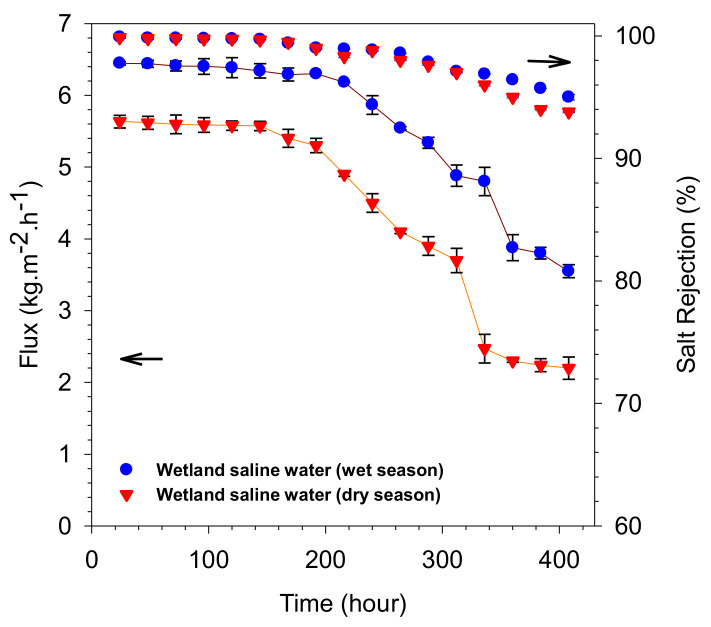
Water fluxes and salt rejection of the silica membrane as the time function over 400 h in wetland saline water at room temperature 25 °C. The wet and dry seasons had 2.7 and 3.3% salts concentrations, respectively.

The long term stability is one major concern of the membrane pervaporation performance. After demonstrating a good pervaporation performance in the short-term experiments, long-term performance was evaluated to gauge the stability of the developed membrane. Figure 6 shows water flux and salt rejection during the long-term wetland saline water desalination. The water flux of wetland saline water during the wet season was 5–10% higher than of at the one obtained during the dry season. It could be attributed to higher salt concentration during the dry season and higher NOM content. Both water flux and salt rejection declined slowly starting from 170 h for the wet season (5.8 kg·m^−2^·h^−1^, 98%) and 140 h for the dry season (5.2 kg·m^−2^·h^−1^, 99%). The sharp declines happened after 360 h (4.8 kg·m^−2^·h^−1^, 97%) for the wet season and 340 h (4.3 kg·m^−2^·h^−1^, 98%) for the dry season. The flux decline for the dry season was faster than the wet season, which can be attributed to higher salt and NOM contents.

Desalination performance in Figure 6 was better than our earlier work [5]. In the previous work, the highest water flux of wetland saline water was only 1.7 kg m^−2^ h^−1^ with 99% of salt rejection during the first 100 h of operation. The water flux then declined sharply to 1.2 kg m^−2^ h^−1^ after 250 h with the salt rejection of merely 75%. Improvements were obtained in this work, with higher water flux, which could be attributed to the change of the fabrication method through the direct deposition of the active layer onto the alumina substrate. This finding concludes that the interlayer-free silica membrane with 4 coating layers was better than the 2 layers within the pore network of the silica matrices.

Figure 6 shows stable performance was obtained for up to 400 h. It was better than a previous work employing a membrane prepared with a similar RTP calcination method [27]. The water fluxes were 2.32 to 1.45 kg/m^−2^·h^−1^ and stable for 300 h. After 350 h the membrane failed due to pore wetting. The employed dual catalysts could contribute to the excellent stability in this work. The micropores and mesopores pores contributed to high performance and good stability. Therefore, RTP can be considered an efficient method in terms of time and costs and offering good performance [14,27,49,50].

The pore-blocking caused by NOM typically lowered the water flux and increased salt rejection. NOM existed in molecular weights (MW) of <1 kDa, 3–10 kDa, 10–30 kDa, and >30 kDa, Song, et al. [51]. If compared to Na^+^ ions (0.022 kDa), Cl^−^ ions (0.035 kDa) and water (0.018 kDa), NOM have much higher MWs and larger sizes. NOM compounds had a molecular size of 5–200 nm [41] higher than the average pore size of the developed membrane (Table 1). Fractionation of NOM revealed their sizes of 5–700 nm [52].

**Figure 7:**
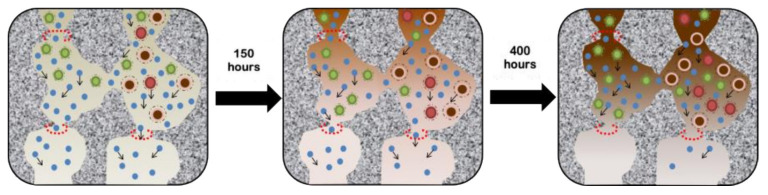
Illustration of pore collapse in silica membrane during the diffusion of water in wetland saline water desalination.

Figure 7 illustrates the transport of NOM in membrane pores. The developed membrane had micro and mesopores. NOM could not pass the micropores and clogged the pore, reducing the water flux and increasing the rejection of salts. The stability results of the pervaporation performance (Figure 6) suggested that the silica membrane matrices underwent pore structural changes causing the pore blockages. During the first 150 h of operation, salt was trapped in the mesopores of the silica matrices, and the water flux and salt rejection could be highly maintained. Afterward, the NOM content influenced the pores until 400 h of operation. After that, salt was trapped in the silica mesopores, and water molecules were restricted to pass through the pores due to high NOM concentration.

**Figure 8:**
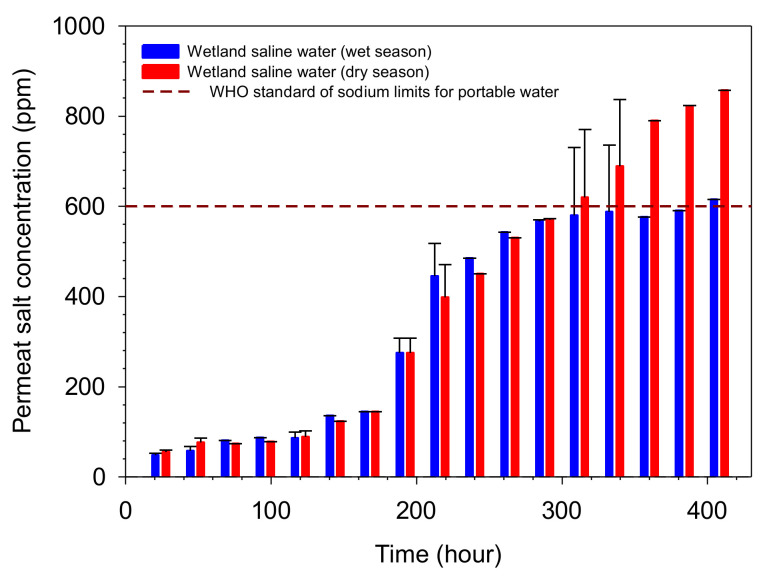
Permeate salt concentration of wetland saline water desalination as a function of exposure time at 25 °C.

The quality of permeate from pervaporation of wetland saline water compared to WHO standard for drinking water is shown in Figure 8. The salt concentration of the permeate met the WHO standard. The permeate quality of the wetland saline water during the wet season was worse than the dry season after 300 h operation. Consequently, the salts concentration of the permeate for the wet season did not meet the WHO standard after 300 h operation.

The developed interlayer-free silica membrane could effectively treat wetland saline water. The high concentration of NOM reduced the water flux over time, as reported earlier [53]. In previous work, membrane technology was applied for peat water with low salt concentration [43,48,53]. Wetland saline water desalination is a new challenge to membrane application due to the high NOM content and the saline conditions. However, the silica membrane in this work showed excellent performance and relatively stable high salt and NOM rejection for wetland saline water application.

## 4. Conclusions

This study demonstrated that the combination of dual catalyzed and RTP methods resulted in highly stable interlayer-free mesoporous silica membranes suitable for wetland saline water pervaporation. The highest water fluxes and salt rejections were 6.5 and 6.9 kg·m^−2^·h^−1^, and salt rejections of 86 and 96% for wetland saline water and seawater, respectively. Excellent long-term stability was demonstrated for 400 h for real wetland saline water desalination. The water flux and salt rejection were 4.8 kg·m^−2^·h^−1^, and 97% for the wet season and 4.3 kg·m^−2^·h^−1^, and 98% for the dry season, respectively. The decline in flux and salt rejection were attributed to the blocking of hydrated salt ions and NOM that constricted the membrane pores.

## Data Availability

Not applicable.
